# Characterization of New *Tropicoporus* Species (Basidiomycota, Hymenochaetales, Hymenochaetaceae) Discovered in Tamil Nadu, India

**DOI:** 10.3390/biology13100770

**Published:** 2024-09-27

**Authors:** Elangovan Arumugam, Ramesh Murugadoss, Sugantha Gunaseelan, Samantha C. Karunarathna, Abdallah M. Elgorban, Pabulo Henrique Rampelotto, Malarvizhi Kaliyaperumal

**Affiliations:** 1 Centre for Advanced Studies in Botany, University of Madras, Guindy Campus, Chennai 600025, Tamil Nadu, India; elangovanaru11@gmail.com (E.A.); rameshmurugadoss@gmail.com (R.M.); suganthagunaseelan@gmail.com (S.G.); 2 Center for Yunnan Plateau Biological Resources Protection and Utilization, College of Biological Resource and Food Engineering, Qujing Normal University, Qujing 655011, China; 3 National Institute of Fundamental Studies (NIFS), Kandy 20000, Sri Lanka; 4 Department of Botany and Microbiology, College of Science, King Saud University, Riyadh 11451, Saudi Arabia; 5 Bioinformatics and Biostatistics Core Facility, Institute of Basic Health Sciences, Federal University of Rio Grande do Sul, Porto Alegre 91501-970, Brazil

**Keywords:** new taxa, Eastern Ghats, Hymenochaetaceae, phylogeny, taxonomy, white rot fungi

## Abstract

**Simple Summary:**

The extensive field work study between 2020 and 2023 led to the discovery of three new species of wood-inhabiting fungi; *Tropicoporus pannaensis*, *Tropicoporus subindicus*, and *Tropicoporus xerophyticus* from the southern part of India. The detailed descriptions and illustrations along with molecular support are presented. These discoveries may contribute to our understanding of species diversity and ecology, ultimately benefiting the society by informing the conservation efforts and exploring the potential biomolecules.

**Abstract:**

This study aimed to investigate the morphological characteristics and phylogenetic relationships of three new species of *Tropicoporus* from the southern parts of India. The analyses of the ITS and nLSU regions revealed the novelty of these species, which have been named *T. pannaensis*, *T. subindicus*, and *T. xerophyticus*. All three species possess pileate basidiomes, a monomitic hyphal system in the context, and the presence of cystidioles and setae. However, they differ significantly in their phylogenetic placements and other morpho-taxonomic features. *Tropicoporus pannaensis* is characterized by a meagrely ungulate basidiome, indistinct zones, and an obtuse margin. *Tropicoporus subindicus* has a triquetrous basidiome and a radially cracked, crusted pileal surface with an acute margin, while *T. xerophyticus* is distinguished by an imbricate, perennial basidiome with an abundantly warted pileal surface. A phylogenetic tree is provided to show the placement of the three new species, along with detailed descriptions and illustrations. Additionally, a key for the identification of the Asian species of *Tropicoporus* is presented.

## 1. Introduction

*Inonotus* P. Karst. represents the largest and most challenging heterogeneous genus within the family Hymenochaetaceae. Segregating species within this genus has proven to be a persistent challenge for mycologists, as the traditional characteristics commonly used to delineate taxa are often inconclusive. The complex taxonomic history and the overlapping morphological features exhibited by many *Inonotus* species have made it difficult to establish clear and reliable species boundaries using only conventional identification methods. Additionally, intergeneric separation was known to be a polyphyletic origin, as *Inonotus* s.l. accommodated several homogeneous subgroups that are evident within [1,2]. Several molecular systematics, especially from the generated nLSU rDNA sequence data, were used to delimit *Inonotus* s.l. into four relatively smaller natural genera of monophyletic origin along with *Inonotus* s.s. [1]. As more molecular data were generated from East Asian and Mesoamerican origin, nearly 15 species were grouped under the *Inonotus linteus* complex [3,4]. Subsequently, morphological and phylogenetic analyses segregated *I. linteus* complex into *Tropicoporus* and its close evolutionary ally *Sanghuangporus* [5]. *Tropicoporus* is currently identified by the presence of resupinate, effused-reflexed to pilear surface, annual to perennial basidiomes with a homogeneous to duplex context, mono-dimitic or strictly dimitic hyphal system, hymenial setae, and basidiospores that are yellowish and have walls that range from slightly thin to thick [5]. Since then, 15 species were added under *Tropicoporus. T. angustisulcatus*, *T. boehmeriae*, *T. drechsleri*, *T. excentrodendri*, *T. flabellatus*, *T. guanacastensis*, *T. hainanicus*, *T. lineatus*, *T*. *minus*, *T. nullisetus*, *T. ravidus*, *T. stratificans*, *T. substratificans*, *T. tenuis*, and *T. texanus* [5,6,7,8,9,10,11]. Hymenial setae was reported to be absent in *T. nullisetus* alone [11]. According to the MycoBank database, as of September 2024, the genus *Tropicoporus* has 56 recognized species. Of these, 23 are newly described species, while 33 represent new taxonomic combinations. However, the phylogenetic relationships among *Tropicoporus* species and their geographical distribution patterns remain uncertain. To better understand the species circumscription and evolutionary history of this genus, additional systematic sampling and examination of specimens from the paleotropical region and other tropical and temperate Asian countries is required. Such comprehensive taxonomic and phylogenetic investigations will help elucidate the true diversity and biogeography of the genus *Tropicoporus*.

Earlier, our team reported the discovery of seven new species of *Tropicoporus* from the southern regions of the India. These species include *T. cleistanthicola*, *T. indicus*, *T. natarajanii*, *T. pseudoindicus*, *T. subramanii*, *T. tamilnaduensis,* and *T. maritimus* [12,13,14]. In the present study, we report the descriptions, illustrations, and phylogenetic analysis results for three novel species of *Tropicoporus* discovered in the state of Tamil Nadu, located in the southern region of India. The detailed morphological characterization and the robust phylogenetic placement of these three new taxa contribute to the growing knowledge of the diversity and evolutionary relationships within the genus *Tropicoporus*, particularly in the understudied mycobiota of the Indian subcontinent.

## 2. Materials and Methods

### 2.1. Sample Collection and Macro- and Micro-Taxonomic Characteristic Analyses

The basidiome samples examined in this study were collected during field surveys conducted between 2020 and 2023. The collection sites included the Veerappanur Reserve Forest in the Jawadhu Hills (coordinates: 12°54′24.9″ N, 78°87′75.6″ E), the Pennaiyar Reserve Forest in Sathanur (coordinates: 12°12′20.6172″ N, 78°53′20.2632″ E), both located within Thiruvannamalai District, and the Karaikudi (coordinates: 10°04′12.00″ N, 78°46′48.00″ E) in Sivagangai District, Tamil Nadu, India.

Morphological characteristics, including size, shape, annual or perennial, colour, texture, and margin (acute or obtuse) of basidiomes, were examined in fresh samples. Colour descriptions were based on the Methuen handbook [15]. The xanthochoric reaction in the context tissue (tissue turning permanently dark brown/black with a drop of KOH solution) was noted for fresh specimens. Other characteristic features like context (colour, homogenous, duplex with/without blackline), tube layer (length, colour, stratification, with context or not), and pores (shape and numbers per mm) were recorded.

For analyzing microscopic characteristics, tissues from dried basidiomes were taken by free-hand sections and mounted in sterile distilled water, 5% KOH solution (*w*/*v*), cotton blue (CB), and Melzer’s reagent (IK). The basidiospores, cystidiole, basidiole, and basidia were observed using phloxine stain. The microscopical features were photographed and illustrations were made as mentioned elsewhere [13]. The mean length and width of the basidiospores, their Q values (derived from an average of 30 basidiospores), and other abbreviations were used as mentioned earlier [13]. The basidiomes were deposited in the herbarium of Madras University Botany Laboratory (MUBL), Centre for Advanced Studies in Botany, University of Madras, Chennai-600 025, Tamil Nadu, India.

### 2.2. PCR Amplification and Phylogenetic Analyses

DNA was extracted from 50 mg of mycelium following the protocol described elsewhere [16] and was modified later [17]. The primers ITS1/ITS4 and LR0R/LR7 were used to amplify the ITS and LSU of nuclear ribosomal DNA region with the recommended thermal conditions [18,19]. The PCR products were then quantified and sequenced at Eurofins Genomics India (Karnataka, India).

Eighteen sequences were generated from the ITS and nLSU region and deposited in GenBank (Table 1). For the phylogenetic analyses, additional sequences from 71 taxa (60 nLSU and 70 ITS sequences), including *Fulvifomes, Inocutis, Inonotus, Phellinus, Phylloporia, and Sanghuangporus*, with an emphasis on *Tropicoporus*, were retrieved from GenBank (NCBI), along with *Fomitiporella caryophylli* (CBS 448.76) and *F. neoarida* (URM 80362) as outgroup (Table 1). To improve alignment similarity, the ITS and nLSU sequences were manually modified after being individually aligned in MEGA X v10.0.2 [20]. Using raxmlGUI 2.0 [21] and MrBayes 3.2.7a [22], respectively, the best-fit evolutionary model found by jModelTest 2.1.10 [23] was employed for the maximum likelihood (ML) analysis and Bayesian inference (BI) analysis. Bayesian inference was performed using two independent runs of six chains of Metropolis-coupled Markov chain Monte Carlo reconstructions for 2,000,000 generations, with tree samples obtained every 100 generations. The final sequence alignment was submitted to TreeBase (submission ID 30913; www.treebase.org).

## 3. Results

### 3.1. Phylogenetic Analyses

**Figure 1:**
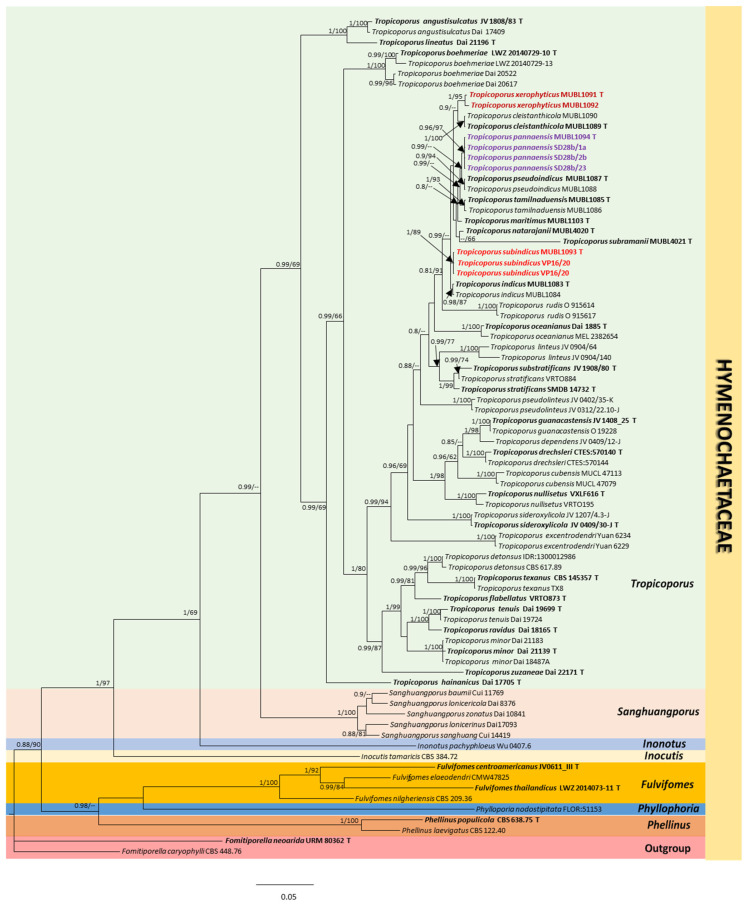
Molecular phylogeny of the three new *Tropicoporus* species from India inferred through a Bayesian analysis of the combined ITS and LSU sequence data. The phylogenetic tree presented shows the placement of the novel taxa in relation to other known *Tropicoporus* species. The numbers indicated at the nodes represent the Bayesian posterior probabilities and bootstrap support values, with only those equal to or above 0.8 and 60%, respectively, being displayed. The type specimens are shown in bold, while the new *Tropicoporus* species are highlighted in colour and presented in bold text.

The similarity ratio of BLAST analyses for ITS and nLSU sequences of the three new species from India are summarized in Supplementary Tables S1 and S2. The total number of characters in the concatenated nLSU and ITS dataset is 1972 (1119 for nLSU and 853 for ITS), of which 1038 were constant, 833 variable, and 618 parsimony-informative. The maximum likelihood (ML) trees were constructed using raxmlGUI 2.0 [21] using the best-fit evolutionary model (GAMMA+P-Invar Model), which was estimated by jModelTest 2.1.10 [23] using 1000 rapid bootstrap inferences (BS). After 2,000,000 generations of Bayesian analysis, the average standard deviation was 0.009. A consistent tree topology was demonstrated by the phylogenetic tree built using ITS and LSU ( Supplementary Figures S1 and S2, respectively). Figure 1 shows the phylogenetic tree produced from the combined ITS and LSU datasets. Phylogenetic analyses inferred from ITS and nLSU reveal that our three new species form a sister clade with *T. rudis* (91% MLBS and 0.81 BI). Our three novel species clustered with allied Indian *Tropicoporus* taxa published earlier (0.99 BPP) and has a mono-dimitic hyphal system [12,13,14].

### 3.2. Taxonomic Characters of the Three New Species of Tropicoporus

***Tropicoporus pannaensis****S. Gunaseelan* & *M*. *Kaliyaperumal* sp. nov.

**Figure 2:**
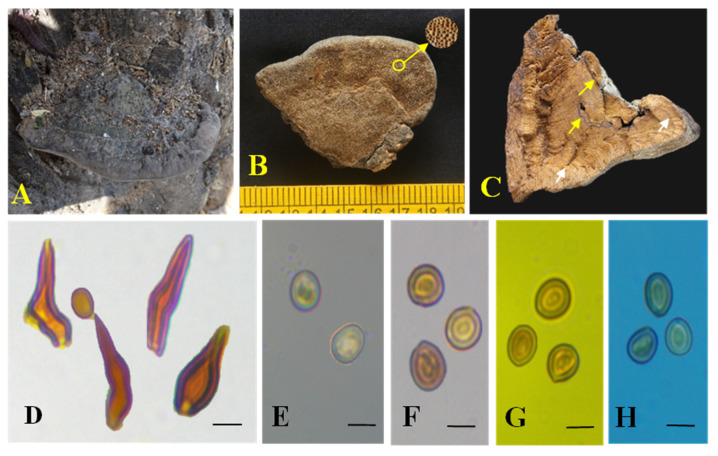
*Tropicoporus pannaensis* (MUBL1094 holotype). (**A**) Holotype basidiomes. (**B**) Pore surface with enlarged pores. (**C**) Cross-section of a basidiome; yellow arrow indicates duplex context with blackline and white arrow indicates stratified tubes. (**D**) Hymenial setae. (**E**) Basidiospores in water. (**F**) Basidiospores in KOH. (**G**) Basidiospore in Melzer’s reagent. (**H**) Basidiospores in cotton blue. Scale bars: 5 µm (**D**–**H**).

**Figure 3:**
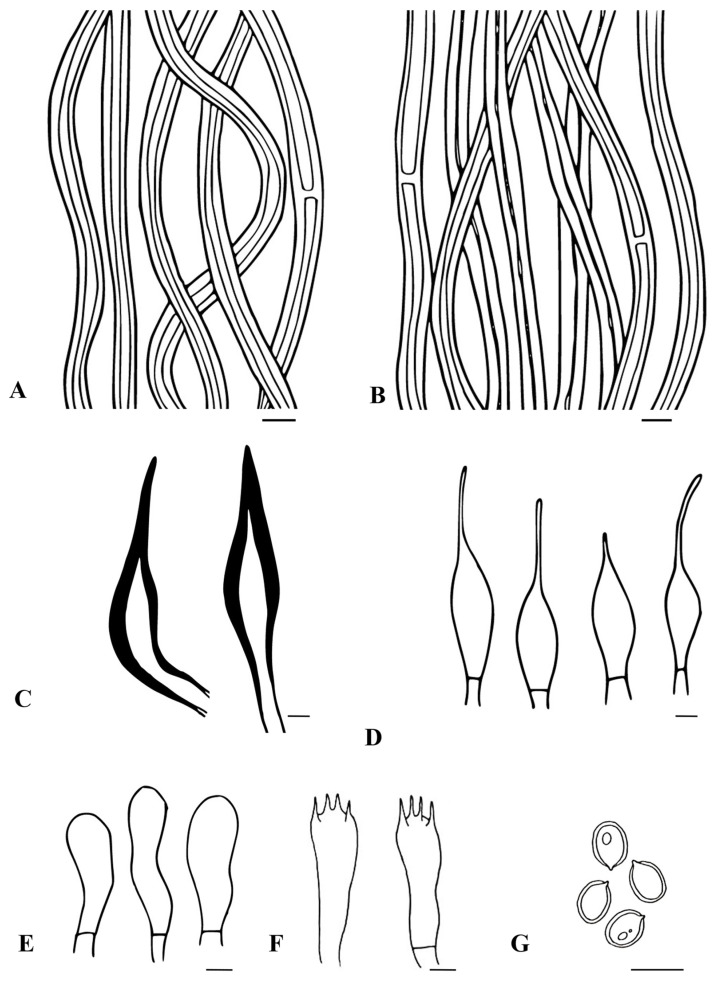
Microscopic structures of *Tropicoporus pannaensis* (from the Holotype). (**A**) Hyphae from context. (**B**) Hyphae from trama. (**C**) Hymenial setae. (**D**) Cystidioles. (**E**) Basidioles. (**F**) Basidia. (**G**) Basidiospores. Scale bars: 5 µm.

Mycobank no: MB850137; Figure 2 and Figure 3

*Etymology:* The species epithet “*pannaensis*” refers to the collection locality (Pennaiyar River, Sathanur Dam)

*Typification*: INDIA, Tamil Nadu, Thiruvannamalai District, Sathanur Dam, (12°15′60.46″ N, 78°96′05.86″ E), on the living tree (*Prosopis juliflora*), 29 January 2023, Sugantha Gunaseelan, SD28B (MUBL1094, Holotype).

*GenBank numbers:* ITS: OR515276; LSU: OR515277

*Diagnosis: Tropicoporus pannaensis* is characterized by applanate to meagrely ungulate, glabrous, zonate pilear surface, obtuse margin, duplex context with blackline, pores 4–7/mm, monomitic hyphal system in context, presence of cystidioles, and subglobose to broadly ellipsoid basidiospores measuring 5.3–5.8 × 4.5–5.3 μm.

*Description:* Basidiomes perennial, pileate, woody, light when fresh, hard when dry, lacks odour or taste. Pilei dimidiate, applanate to meagrely ungulate, projecting up to 5 cm, 6.7 cm wide, and 2.4 cm thick near attachment. Pileal surface brown (6E4) to greyish-brown (6E3), glabrous, zonate, tuberculate near the attachment. Margin obtuse, up to 3 mm thick, greyish-brown (6E3). Pore surface greyish-brown (6D3) and light brown (6D4), glancing. Pores round to angular, 4–6/mm. Context zonate, duplex with black line, brown (6D6), up to 2.4 cm thick. Tubes up to 2.5 cm long, stratified, and each stratum up to 2.4 mm, brown (6D6).

Hyphal system tissue darkening with KOH without hyphal swelling; monomitic in the context and dimitic in the trama; context: generative hyphae, thin- to thick-walled, hyaline to golden brown, occasionally branched with simple septate, 2–5 μm diam. Trama: generative hyphae, dominant, thin to thick-walled, hyaline to pale yellow, occasionally branched with septate, 2–4.5 μm diam; skeletal hyphae, thick-walled with narrow to wide lumen, yellowish-brown, unbranched, aseptate, 2–3 μm diam. Hymenial setae thick-walled, ventricose to subulate with a sharp and obtuse tip, dark brown, 14.2–28.4 × 3.8–5.6 μm. Cystidioles hyaline, thin-walled, ventricose to fusoid with elongated tapering apical portion, 9.8–23.73 × 3.8–5.2 μm. Basidia clavate to subclavate, 7.2–12 × 3–5.2 μm, with four sterigmata. Basidiole clavate, 4–11.4 × 3.1–5.2 μm. Basidiospores smooth, broadly ellipsoid to subglobose, thick-walled, pale yellow to golden yellow in water, turning golden yellow to brown in KOH, (5.3–) 5.5–5.8 × (4.5–) 4.7–5 (–5.3) μm (n = 30), Q = 1.11 (Q range 1.05–1.18), CB^−^, IKI^−^.

*Habitat and distribution:* Basidiomes are found on living trees of *Prosopis juliflora* (Fabaceae) distributed in Jawadhu Hills, Thiruvannamalai District, Tamil Nadu, India.

***Tropicoporus subindicus****R. Murugadoss, E. Arumugam & M. Kaliyaperumal* sp. nov.

**Figure 4:**
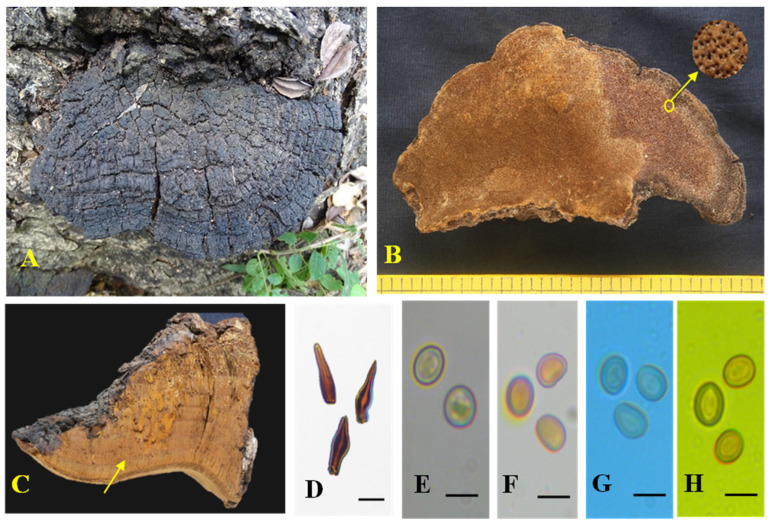
*Tropicoporus subindicus* (MUBL1093 Holotype) (**A**) Basidiome (Holotype). (**B**) Pore surface with enlarged pores. (**C**) Cross-section of a basidiome; yellow arrow represents stratified tubes. (**D**) Hymenial setae. (**E**) Basidiospores in water. (**F**) Basidiospores in KOH. (**G**) Basidiospores in cotton blue. (**H**) Basidiospores in Melzer’s reagent. Scale bars: (**D**–**H**) = 5 µm.

**Figure 5:**
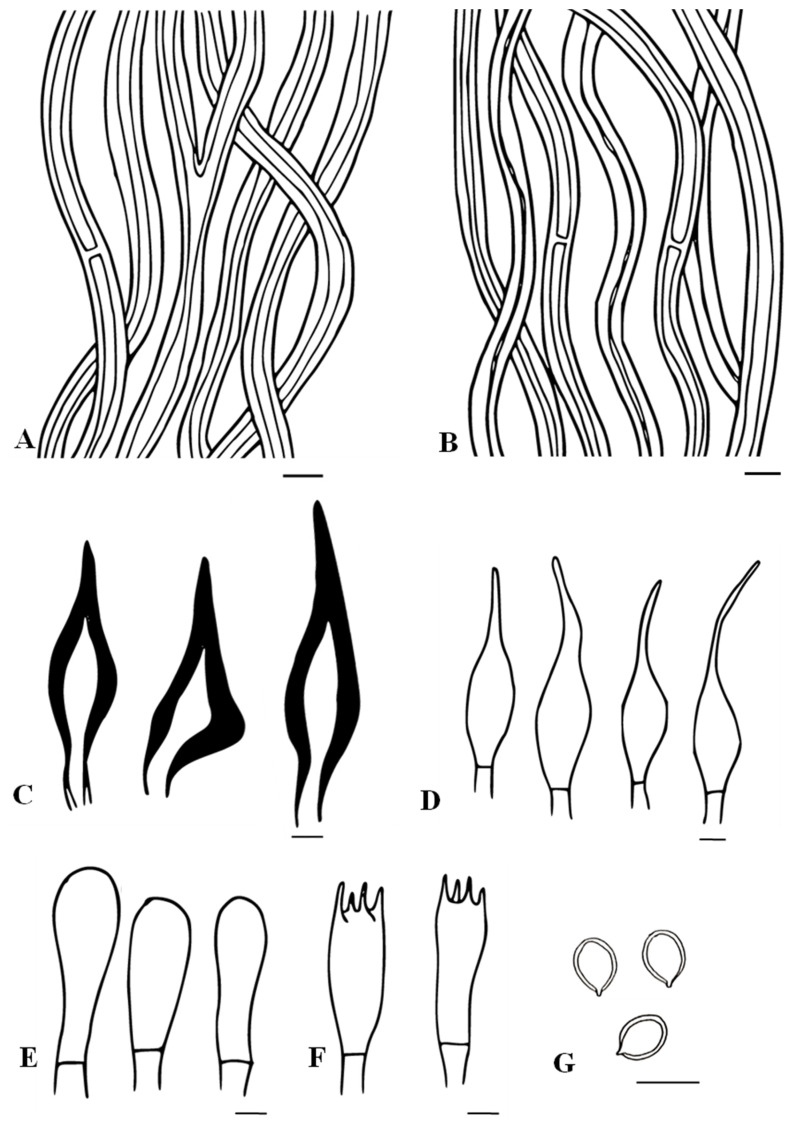
Microscopic structures of *Tropicoporus subindicus* (from the Holotype). (**A**) Hyphae from context. (**B**) Hyphae from trama. (**C**) Hymenial setae. (**D**) Cystidioles. (**E**) Basidioles. (**F**) Basidia. (**G**) Basidiospores. Scale bars: 5 µm.

Mycobank: MB850136; Figure 4 and Figure 5

*Etymology:* The term “subindicus” refers to the tight evolutionary relationship between the species and *Tropicoporus indicus*.

*Typification:* INDIA, Tamil Nadu, Thiruvannamalai District, Veerapanur Reserve Forest, Jawadhu Hills (12°61′95.61″ N, 78°92′89.46″ E), on dead wood, 28 January 2020, Ramesh Murugadoss, VP16 (MUBL1093, Holotype)

*GenBank numbers:* ITS: OR519719; LSU: OR519722

*Diagnosis: Tropicoporus subindicus* is characterized by ungulate to triquetrous basidiome with concentrically zonate and sulcate, pilear surface radially cracked, context homogenous with monomitic hyphal system, acute margin, presence of cystidioles and setae, and subglobose to broadly ellipsoid basidiospores measuring 5–5.5 × 4.3–5.5 μm.

*Holotype:* MUBL1093

*Description:* Basidiomes perennial, pileate, woody, light in weight, hard when dry, without odour or taste. Pilei dimidiate, ungulate to triquetrous, projecting up to 8.3 cm, 20.4 cm wide, and 5.6 cm thick near attachment. Pileal surface greyish-brown (6F3) to brownish-grey (7F2), radially cracked, concentrically zonate and sulcate with crust. Margin acute, incurved towards pilear surface, >1 mm thick, dark brown (6F6). Pore surface dark brown (7E5) and brownish-orange (5C6), glancing. Pores round to angular, 4–6/mm. Context homogenous, brown (6E6), up to 1 mm thick. Tubes up to 5.5 cm long, stratified, each stratum up to 4.4 mm, light brown (6D6).

Hyphal system tissue darkens in KOH without hyphal swelling; monomitic in the context and dimitic in trama; generative hyphae, thin- to thick-walled, hyaline to golden yellow, rarely branched, simple septate, 2–5 μm diam. Trama: generative hyphae, dominant, thin- to thick-walled, hyaline to yellowish, occasionally branched, septate, 2–4.5 μm diam.; skeletal hyphae, thick-walled with narrow to wide lumen, yellowish-brown, unbranched, aseptate, 2–3 μm diam. Hymenial setae thick-walled, ventricose to subulate with a sharp and blunt tip, dark brown, 6.5–27.5 × 2.5–5.5 μm. Cystidioles thin-walled, hyaline, ventricose to fusoid with elongated tapering apical portion, 10–18 × 3–5 μm. Basidia clavate to broadly clavate, 8–10 × 3–5 μm, with four sterigmata. Basidiole clavate, 3.6–12 × 3.3–5 μm. Basidiospores broadly ellipsoid to subglobose, thick-walled, pale yellow in water, turning golden yellow to brown in KOH, CB^−^, IKI^−^, (5–) 5.3–5.5 × (4.3–) 4.5–4.8 (–5.5) μm (n = 30), Q = 1.10 (Q range 1–1.17).

*Habitat and distribution:* Basidiomes found on dead wood, Jawadhu Hills, Thiruvannamalai District, Tamil Nadu, India.

***Tropicoporus xerophyticus****E. Arumugam & M. Kaliyaperumal* sp. nov.

**Figure 6:**
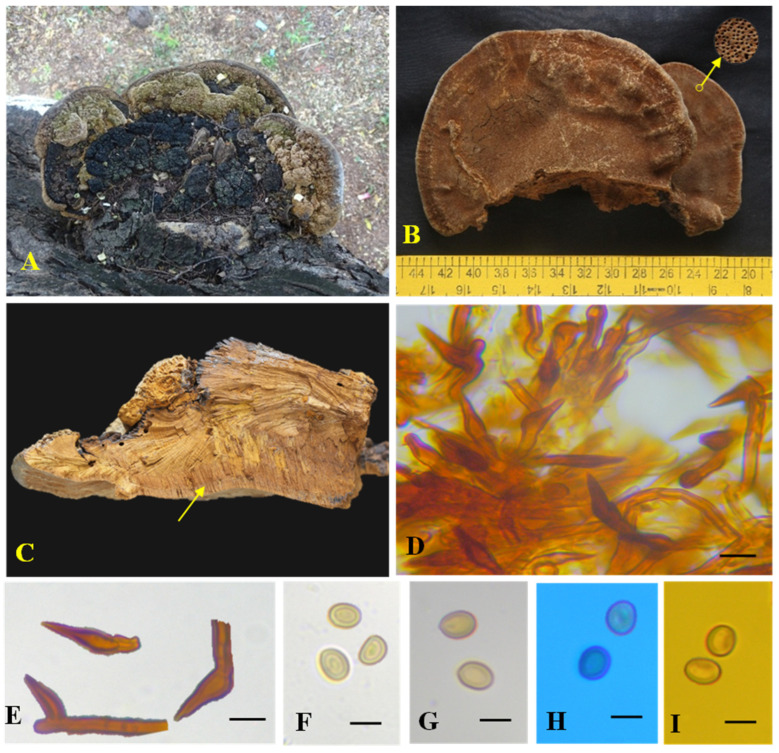
*Tropicoporus xerophyticus* (MUBL1091 holotype). (**A**) Basidiomes (Holotype). (**B**) Pore surface with enlarged pores. (**C**) Cross-section of a basidiome; yellow arrow indicates stratified tubes. (**D**,**E**) Hymenial setae. (**F**–**I**) Basidiospores: (**F**) Basidiospores in water. (**G**) Basidiopores in KOH. (**H**). Basidiopores in cotton blue. (**I**) Basidiopores in Melzer’s reagent. Scale bars: (**D**–**I**) = 5 µm.

**Figure 7:**
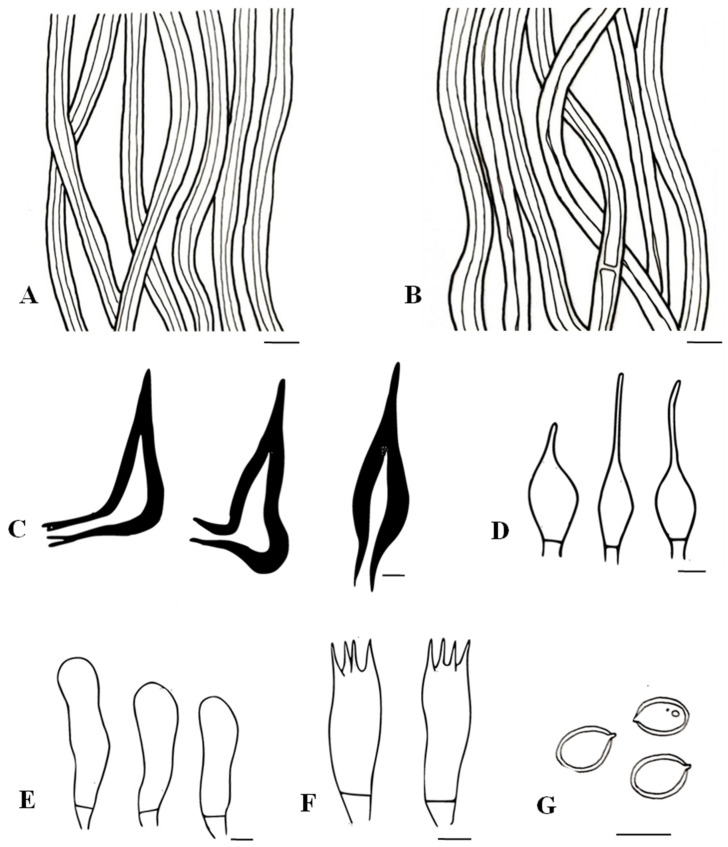
*Tropicoporus xerophyticus* (MUBL1091 from the Holotype). (**A**) Contextual hyphae. (**B**) Tramal hyphae. (**C**) Hymenial setae. (**D**) Cystidioles. (**E**) Basidioles. (**F**) Basidia. (**G**) Basidiospores. Scale bars = 5 µm.

*MycoBank*: MB850135; Figure 6 and Figure 7

*Etymology*: The term “*xerophyticus*” refers to the dry environmental conditions in which the new species grows.

*Typification:* INDIA, Tamil Nadu, Karaikudi District, Tamil Nadu, (10°08′70.56″ N, 78°79′39.40″ E), on living angiosperm tree (*Acacia arabica*), 23 January 2023, Elangovan Arumugam, ALP18 (MUBL1091, Holotype).

*GenBank numbers:* ITS: OR515186; LSU: OR515187

*Diagnosis: Tropicoporus xerophyticus* is characterized by perennial, imbricate, broadly zonate, sulcate, deep fissures at maturity, warted, obtuse to round margin, homogenous context with monomitic hyphal system, presence of cystidioles and setae, and subglobose to broadly ellipsoid basidiospores measuring 4.5–5.5 × 4.2–5 μm.

*Description:* Basidiomes perennial, solitary, pileate, light when fresh, hard when dry, lacks odour or taste. Pilei dimidiate, imbricate, projecting up to 12 cm, 23 cm wide and 5 cm thick near attachment. Pilear surface greyish-brown (7F3) to dark grey (1F1), frequently warted towards margin, broadly zonate, sulcate, distinctly cracked with deep fissures at maturity. Margin yellowish-brown (5D5), obtuse to round, 1.8 cm thick. Pore surface dark brown (6F6). Pores round to angular, 3–6/mm; context brown (6D6), homogenous, up to 2.5 cm thick. Tubes brown (6E7), 2.4 cm, with intermittent context, stratified, each stratum up to 3.8 mm.

Hyphal system tissue darkens with KOH without hyphal swelling, mono-dimitic; context: generative hyphae, thin- to thick-walled, hyaline to golden yellow, occasionally branched, simple septate, 2–5 μm diam. Trama: generative hyphae, dominant, thin- to thick-walled, hyaline to yellowish, occasionally branched, septate, 2–4 μm diam.; skeletal hyphae, thick-walled with narrow to wide lumen, yellow to yellowish-brown, unbranched, aseptate, 2–3.2 μm diam. Hymenial setae thick-walled, ventricose to subulate with a sharp to blunt tip, dark brown, 12–27.5 × 4.7–5.5 μm. Cystidioles hyaline, thin-walled, fusoid, with elongated tapering apical portion 10–15.5 × 2.2–3 μm. Basidia clavate to subclavate, 8–10 × 3–5 μm, with four sterigmata. Basidiole clavate, 3–10 × 3.5–5 μm. Basidiospores smooth, subglobose to broadly ellipsoid, fairly thick-walled to thick-walled, pale yellow to golden yellow in water, turning golden yellow to brown in KOH, CB^−^, IKI^−^, (4.5–) 4.8– 5 (–5.5) × (4.2–) 4.5–4.7 (–5) μm (n = 30), Q = 1.08 (Q range 1.06–1.17).

*Additional material examined:* INDIA, Tamil Nadu, Karaikudi District, Tamil Nadu, 10°08′69.40″ N, 78°79′37.20″ E, on living angiosperm tree (*Acacia arabica*), 23 January 2023, Dr. K. Malarvizhi, ALP33 (MUBL1092, Paratype).

*GenBank numbers:* ITS: OR515255; LSU: OR515267

*Habitat and distribution:* Basidiomes are found on living trees of *Acacia arabica* (Fabaceae) distributed in Karaikudi, Sivagangai District, Tamil Nadu, India.

## 4. Discussion

In addition to comprehensive traditional taxonomic studies, phylogenetic analyses resolved the uncertainty in *Inonotus* s.l. [2,24,25] and delimited *I. linteus* complex into *Inonotus* s.str., *Tropicoporus*, and *Sanghuangporus* [3,4,5]. India harbours nearly four hotspots of rich vegetation; however, the members of Hymenochaetoid fungi were explored from northern parts of India and illustrated only by conventional methods [26,27,28]. In Southern India, in continuation with our earlier report from Eastern Ghats (a fragmented mountain range of lower elevation with disturbed vegetation) [13], we report two additional species of *Tropicoporus*. The ML and Bayesian trees depicted in this paper (Figure 1) validate the topology and are similar to previous reports [10,13,14].

*Tropicoporus xerophyticus* formed a sister clade with *T. cleistanthicola* (0.9 BPP). While analyzing the morphology of *T. xerophyticus* and *T. cleistanthicola*, the former significantly differs in the imbricate basidiome, which is broadly zonate with abundant warts; has an obtuse margin; and is distinctly cracked with deep fissures and larger pores (3–6/mm), whereas *T. cleistanthicola* has a pileate with an uncracked basidiome, is narrowly zonate, and has an acute margin and smaller pores (5–7/mm) [13]. *Tropicoporus xerophyticus* is phylogenetically distinct from other Indian allied taxa, namely *T. pseudoindicus, T. tamilnaduensis, T. maritimus, T. natarajanii*, and *T. subramanii* but are similar in mono-dimitic hyphal system alone and significantly varies in other morpho-taxonomic characters. *Tropicoporus xerophyticus* and *T. subramanii* resemble each other by having a pileate with a cracked basidiome, larger pores, and a hyphal system, but the former lacks a crust in the pileus and has an obtuse margin and smaller spores [12]. *Tropicoporus xerophyticus* and *T. maritimus* are congruous, having a pileate and broadly zonate basidiome but our new species greatly differ by having an imbricate basidiome that is cracked with deep fissures, a homogenous context, and an obtuse to round margin [14]. *Tropicoporus xerophyticus* and *T. pseudoindicus* resemble by having a cracked basidiome and an obtuse margin but the former significantly differs from the latter by having an imbricate and warted basidiome, larger pores (3–6/mm), and homogenous context; *T. pseudoindicus* was reported to have larger pores (6–8/mm) and a duplex context [13]. *Tropicoporus xerophyticus* varies from *T. tamilnaduensis* in having an imbricate, warted basidiome with larger pores (3–6/mm) and smaller cystidioles [13]. Though *T. xerophyticus* and *T. rudis* have a homogenous context and mono-dimitic hyphal system, the former varies from the *T. rudis* in basidiome characteristics and acyanophilic basidiospore, with the latter species having cyanophilic basidiospores [10]. *Tropicoporus xerophyticus* shares a homogenous context and distinctly cracked basidiomes with *T. pesudolinteus* and *T. sideroxylicola*, but the Indian species differs in other morphological characteristics, like having an imbricate, frequently warted, and broadly zonate basidiome [4]. *Tropicoporus xerophyticus* and *T. stratificans* are similar only in the presence of an intermittent context in the trama and the presence of cystidioles, while the latter has a resupinate and glancing basidiomes, dimitic hyphal system, and smaller basidiospores (3.5–6 × 3–4.5 μm) [7].

*Tropicoporus pannaensis* is phylogenetically distinct from *T. pseudoindicus* (0.99 BI and 57% MLBS) [13]. Morphologically, *T. pannaensis* differs with *T. pseudoindicus* in having an uncracked basidiome, larger pores (4–6/mm), and larger basidiospores (5.5–5.8 × 4.7–5), while *T. pseudoindicus* has a distinctly cracked basidiome, smaller pores (6–8/mm). and smaller basidiospores (4.2–5 × 4–4.5) [13]. *Tropicoporus pannaensis* is similar with *T. tamilnaduensis*, *T. subramanii*, and *T. rudis* by having pileate basidiomes and a hyphal system, but our species has uncracked basidiomes and a duplex context [10,12,13]. *Tropicoporus pannaensis* resembles *T. maritimus* with a few morphological features such as a pileate uncracked basidiome and duplex context, but the former differs in pilear surface characters such as a meagrely ungulate, frequently warted basidiome, tuberculate near attachment, and obtuse margin [14]. *Tropicoporus pannaensis* and *T. natarajanii* are similar by having an uncracked basidiome and a duplex context; however, *T. pannaensis* significantly varies in the glabrous pilear surface, having a broadly zonate, larger setae and smaller basidiospores. However, *T. natarajanii* has a velutinate azonate pileal surface with abundant tuberculate, smaller setae, and larger basidiospores [12]. *Tropicoporus pannaensis* and *T. cleistanthicola* are similar in the zonate basidiome, but our new species differ in having a glabrous basidiome, duplex context, obtuse margin, smaller cystidioles (9.8–23.73 × 3.8–5.2 μm), and larger spores. *Tropicoporus cleistanthicola* has a warted pileal surface, homogenous context, acute margin, larger cystidioles (7–45 × 2–5 μm), and smaller basidiospores (*T. pannaensis* (5.3–) 5.5–5.8 × (4.5–) 4.7–5 (–5.3) μm vs. *T. cleistanthicola* (4.7–) 4.9–5.2 (–5.4) × (4.2–) 4.5–4.7 (–4.9) μm) [13].

*Tropicoporus pannaensis* is different from *T. stratificans* and *T. substratificans* in morphological (applanate to meagrely ungulate basidiome, pores (4–6/mm) and microscopic characteristics (mono-dimitic hyphal system) [7,10]. *Tropicoporus pannaensis* differs from *T. sideroxylicola* by having uncracked and zonate basidiomes, smaller pores, a homogenous context, and a mono-dimitic hyphal system [4].

*Tropicoporus subindicus* is closely clustered with *T. indicus* but *T. subindicus* and these formed a sister to other Indian *Tropicoporus* spp. (BPP 0.99). *Tropicoporus subindicus* differs with *T. indicus* by having a radially cracked basidiome with crust, incurved margin, and is more or less concolorous with pileus colour and smaller basidiospores ((5–) 5.3–5.5 × (4.3–) 4.5–4.8 (–5.5) μm), while *T. indicus* is indistinctly cracked, lacks crust and an entire margin, and has distinctly yellow and larger basidiospores (5–6 × 4.2–4.9 μm) [13].

*T. subindicus* shares few characters with *T. maritimus* in having an acute incurved margin, but the former varies in having an ungulate to triquetrous, radially cracked crusted basidiome and a homogenous context [14]. *Tropicoporus subindicus* and *T. pseudoindicus* has a cracked and sulcate pilear surface (13). However, *T. subindicus* varies by having a concentrically zonate, acute incurved margin, homogenous context, larger pores (4–6/mm), and smaller cystidioles but *T. pseudoindicus* has a broadly zonate, duplex context, acute to obtuse margin, smaller pores (6–8/mm), and larger cystidioles [13]. *Tropicoporus subindicus* shares similar features with *T. subramanii* in having a cracked pilear surface, acute margin, homogenous context, and hyphal system, but our new species differs by having a concentrically zonate pileus, presence of cystidioles, and smaller basidiospores (5–) 5.3–5.5 × (4.3–) 4.5–4.8 (–5.5) μm, while *T. subramanii* has a deeply rimose pilear surface, absence of cystidioles, and larger basidiospores (5–) 5.3–6.4 (–6.7) × (4.5–) 4.8–5 (–5.2) μm [12]. *Tropicoporus subindicus* and *T. natarajanii* are consistent only in the hyphal system but *T. subindicus* significantly differs in morphological characteristics such as a cracked crust, concentrically zonate and sulcate basidiome, acute margin, and homogenous context, while *T. natarajanii* has an uncracked, azonate, abundant tuberculate without a crust pilear surface, obtuse margin, and duplex context [12]. *Tropicoporus subindicus* is similar to *T. cleistanthicola* in having a homogenous context and an acute margin, but *T. subindicus* varies in the pilear surface with a radially cracked crust, concentrically zonate sulcate basidiome, and smaller cystidioles. While *T. cleistanthicola* has an uncracked, narrowly zonate, warted basidiome and larger cystidioles [13]. *Tropicoporus subindicus* is congruous with *T. tamilnaduensis* in having a cracked basidiome, homogenous context, and pores but *T. subindicus* varies by having a concentrically zonate, crust pilear surface, acute margin and smaller cystidioles (10–18 × 3–5) μm, while *T. tamilnaduensis* has broadly zonate obtuse margin and larger cystidioles (10–45 × 2–5) μm [13].

We provide below the key to species of *Tropicoporus* in the Afro-Asian region.

**Key to species of *Tropicoporus* in the Afro-Asian region**
1Basidiomes resupinate to effused-reflexed21Basidiomes distinctly pileate 82Basidiomes annual to biennial32Basidiomes perennial63Basidiospores cyanophilic 
***T. tenuis***
3Basidiospores acyanophilic44Basidiome resupinate to effused reflexed, pileal surface tomentose to hispid basidiospores > 3 μm in length 
***T. excentrodendri***
4Basidiome resupinate, basidiospores < 3 μm in length55Dissepiments lacerate, context layer present between tube layers
***T. hainanicus***
5Dissepiments entire, context layer absent between tube layers
***T. boehmeriae***
6Basidiomes resupinate, cystidioles present 76Basidiomes cushion-shaped, cystidioles absent
***T. ravidus***
7Pores 10–12/mm, basidiospores < 3 μm wide 
***T. minor***
7Pores 6–8/mm, basidiospores > 3 μm wide 
***T. zuzanae***
8Hyphal system strictly dimitic 
***T. lineatus***
8Hyphal system mono-dimitic, dimitic in trama 99Basidiomes uncracked 109Basidiomes cracked to rimose 1310Pilear surface warted; Pores always >5/mm 1110Pilear surface glabrous; Pores < 5/mm1211Pilear surface azonate with warts, obtuse margin, context duplex without blackline 
***T. natarajanii***
11Basidiomes with infrequent warts, acute margin and homogenous context 
***T. cleistanthicola***
12Pilear surface indistinctly zonate, margin obtuse 
***T. pannaensis***
12Pilear surface broadly zonate, margin acute 
***T. maritimus***
13Pilear surface frequently warted with deep cracks at maturity, stratified tube with intermittent context 
***T. xerophyticus***
13Pilear surface cracked, lacks warts, stratified tubes without intermittent context 1414Pilear surface fulvous, velvety and cyanopilous basidiospores 
***T. rudis***
14Pilear surface smooth to glabrous or sulcate and acyanophilous basidiospores 1515Context duplex with black line 
***T. pseudoindicus***
15Context homogenous 16 16Absence of cystidioles 
***T. subramanii***
16Presence of cystidioles 1717Obtuse margin, cystidiole more than 25 µm in length, hymenial setae not exceeding 20 µm in length 
***T. tamilnaduensis***
17Acute margin, cystidiole not exceeding 25 µm in length, hymenial setae more than 20 µm in length 1818Basidiomes with radially cracked, sulcate, crusted pilear surface 
***T. subindicus***
18Basidiomes with glabrous, irregularly cracked pilear surface without crust 
***T. indicus***



## 5. Conclusions

While previous studies on Hymenochaetoid fungi in India focused on the northern regions using only conventional taxonomic methods, this work builds on the authors’ earlier discoveries from the Eastern Ghats, reporting two additional new *Tropicoporus* species from Southern India. The phylogenetic relationships inferred from the ITS and nLSU sequence data place the three novel *Tropicoporus* taxa as a sister clade to *T. rudis*. Detailed morphological comparisons highlight how the new species, *T. xerophyticus*, *T. subindicus*, and *T. pannaensis*, differ from each other and from previously known *Tropicoporus* species in terms of macroscopic features like basidiome characteristics, pore sizes, and microscopic details such as, cystidioles, setae, and basidiospore dimensions. These findings contribute to the understanding of the taxonomic complexity and diversity within the genus *Tropicoporus*, particularly in the understudied mycobiota of southern India.

## Figures and Tables

**Table 1:** Species, strain numbers, geographical locations, and corresponding GenBank accession numbers of the taxa used in this study.

Species	Strain Numbers	Geographical Locations	Accession Numbers	
			ITS	LSU
*Fomitiporella caryophylli*	CBS 448.76	India	AY558611	AY059021
*F. neoarida ^t^*	URM 80362	Brazil	KM211294	KM211286
*Fulvifomes centroamericanus ^t^*	JV0611_III	Guatemala	KX960763	KX960764
*F. elaeodendri*	CMW47825	South Africa	MH599094	MH599134
*F. nilgheriensis*	CBS 209.36	USA	AY558633	AY059023
*F. thailandicus ^t^*	LWZ 2014073-11	Thailand	KR905672	KR905665
*Inocutis tamaricis*	CBS 384.72	-	AY558604	MH872221
*Inonotus pachyphloeus*	Wu 0407.6	Taiwan	KP030785	KP030770
*Phellinus laevigatus*	CBS 122.40	USA	MH856059	MH867554
*P. populicola ^t^*	CBS 638.75	Finland	MH860960	MH872729
*Phylloporia nodostipitata*	FLOR:51153	Brazil	KJ639057	KJ631414
*Sanghuanporus baumii*	Cui 11769	China	MF772784	MF772803
*S. lonicericola*	Dai 8376	China	JQ860308	MF772805
*S. lonicerinus*	Dai 17093	China	MF772788	MF772807
*S. sanghuang*	Cui 14419	China	MF772789	MF772810
*S. zonatus*	Dai 10841	China	JQ860306	KP030775
*Tropicoporus angustisulcatus*	Dai 17409	Brazil	MZ484584	MZ437417
*T. angustisulcatus ^t^*	JV 1808/83	French Guiana	MZ484585	MZ437418
*T. boehmeriae*	LWZ 20140729-13	Thailand	KT223641	MT319394
*T. boehmeriae*	Dai 20522	China	MZ484586	MZ437419
*T. boehmeriae*	Dai 20617	China	MZ484587	MZ437420
*T. boehmeriae ^t^*	LWZ 20140729-10	Thailand	KT223640	MT319393
*T. cleistanthicola*	MUBL1090	India	OR272291	OR272336
*T. cleistanthicola ^t^*	MUBL1089	India	OR272292	OR272337
*T. cubensis*	MUCL 47113	Cuba	JQ860324	KP030777
*T. cubensis*	MUCL 47079	Cuba	JQ860325	KP030776
*T. dependens*	JV 0409/12-J	USA	KC778777	MF772818
*T. detonsus*	CBS 617.89	-	AF534077	AY059037
*T. detonsus*	IDR 1300012986	USA	KF695121	KF695122
*T. drechsleri*	CTES:570144	Argentina	MG242437	MG242442
*T. drechsleri* *^t^*	CTES:570140	Argentina	MG242439	MG242444
*T. excentrodendri*	Yuan 6234	China	KP030791	-
*T. excentrodendri*	Yuan 6229	China	KP030789	-
*T. flabellatus ^t^*	VRTO873	Brazil	MT908376	MT906643
*T. guanacastensis*	O 19228	Costa Rica	KP030794	-
*T. guanacastensis ^t^*	JV 1408_25	Costa Rica	KP030793	KP030778
*T. hainanicus ^t^*	Dai 17705	China	MZ484588	MZ437421
*T. indicus*	MUBL1084	India	OR272294	OR272339
*T. indicus ^t^*	MUBL1083	India	OR272293	OR272338
*T. lineatus ^t^*	Dai 21196	Malaysia	MZ484594	MZ437426
*T. linteus*	JV 0904/64	USA	JQ860322	JX467701
*T. linteus*	JV 0904/140	USA	JQ860323	KP030780
*T. maritimus ^t^*	MUBL1103	India	PP378327	PP378328
*T. minor ^t^*	Dai 21139	China	MZ484592	MZ437424
*T. minor*	Dai 18487A	China	MZ484590	MZ437422
*T. minor*	Dai 21183	China	MZ484593	MZ437425
*T. natarajanii ^t^*	MUBL4020	India	OP003881	-
*T. nullisetus*	VRTO195	Brazil	MN795118	MN812254
*T. nullisetus ^t^*	VXLF616	Brazil	MN795129	MN812261
*T. oceanianus ^t^*	Dai 18859	Australia	PP034280	-
*T. oceanianus*	MEL 2382654	Australia	KP013017	KP013017
***T. pannaensis***	**SD28b/1a**	**India**	**OR520889**	**OR520892**
***T. pannaensis***	**SD28b/2b**	**India**	**OR520890**	**OR520891**
***T. pannaensis***	**SD28b/23**	**India**	**OR520916**	**OR520917**
***T. pannaensis ^t^***	**MUBL1094**	**India**	**OR515276**	**OR515277**
*T. pseudoindicus*	MUBL1088	India	OR272296	OR272341
*T. pseudoindicus ^t^*	MUBL1087	India	OR272295	OR272340
*T. pseudolinteus*	JV 0312/22.10-J	Venezuela	KC778780	-
*T. pseudolinteus*	JV0402/35-K	Venezuela	KC778781	MF772820
*T. ravidus ^t^*	Dai 18165	China	MZ484595	MZ437427
*T. rudis*	O 915614	Rwanda	KP030796	-
*T. rudis*	O 915617	Tanzania	KP030797	MH101016
*T. sideroxylicola*	JV 1207/4.3-J	USA	KC778783	-
*T. sideroxylicola ^t^*	JV 0409/30-J	USA	KC778782	-
*T. stratificans ^t^*	SMDB 14732	Brazil	KM199689	-
*T. stratificans*	VRTO884	Brazil	MN795124	MN812266
***T. subindicus***	**VP16/20**	**India**	**OR520914**	**OR520915**
***T. subindicus***	**VP16/23**	**India**	**OR520912**	**OR520913**
***T. subindicus ^t^***	**MUBL1093**	**India**	**OR519719**	**OR519722**
*T. subramanii ^t^*	MUBL4021	India	OP003882	-
*T. substratificans ^t^*	JV 1908/80	French Guiana	MZ484597	MZ437429
*T. tamilnaduensis*	MUBL1086	India	-	OR272344
*T. tamilnaduensis ^t^*	MUBL1085	India	OR272297	OR272343
*T. tenuis*	Dai 19724	China	MZ484599	MZ437431
*T. tenuis ^t^*	Dai 19699	China	MZ484598	MZ437430
*T. texanus*	TX8	USA	MN108123	MN113949
*T. texanus ^t^*	CBS 145357	USA	NR_168219	NG_068906
***T.*** ***xerophyticus***	**MUBL1092**	**India**	**OR515255**	**OR515267**
***T.*** ***xerophyticus ^t^***	**MUBL1091**	**India**	**OR515186**	**OR515187**
*T. zuzanae ^t^*	Dai 22171	China	PP034282	PP034284

^*t*^ Type materials; Novel *Tropicoporus* spp. from the present study are indicated in bold.

## Data Availability

All holotype and paratype collections of the new species are deposited at Madras University Botany Laboratory (MUBL), Centre for Advanced Studies in Botany, University of Madras, Chennai-600 025, Tamil Nadu, India. The sequences generated during this study are deposited in NCBI GenBank. The ITS and nLSU alignment is deposited in TreeBase.

## Supplementary Materials

The following supporting information can be downloaded at https://www.mdpi.com/article/10.3390/biology13100770/s1, Supplementary Table S1. Similarities of ITS BLAST sequences of three new Tropicoporus species and its related species; Table S2. Similarities of LSU BLAST sequences of three new Tropicoporus species and its related species; Supplementary Figure S1. Molecular Phylogeny of three new Indian *Tropicoporus* species inferred from Bayesian analysis of ITS sequences. The numbers indicated at the nodes represent the Bootstrap values and Bayesian posterior probabilities that equal to or above 60% and 0.9, respectively. The type specimens are in bold; the new *Tropicoporus* spp. are indicated in colour and bold; Supplementary Figure S2. Molecular Phylogeny of three new Indian *Tropicoporus* species inferred from Bayesian analysis of nLSU sequences. The numbers indicated at the nodes represent the Bootstrap values and Bayesian posterior probabilities that equal to or above 60% and 0.9, respectively. The type specimens are in bold; the new *Tropicoporus* spp. are indicated in colour and bold.
