# Supplementary material for: Characterization of New Tropicoporus Species (Basidiomycota, Hymenochaetales, Hymenochaetaceae) Discovered in Tamil Nadu, India
Source: Biology (Basel). 2024 Sep 27;13(10):770. doi: 10.3390/biology13100770 (PMC11504104; doi:10.3390/biology13100770)
Supplement: Supplementary file 1 [file biology-13-00770-s001.zip › Supplementary Tables.pdf]

Table S1. Similarities of ITS BLAST sequences of three new *Tropicoporus* species and its related species.

**Similarity % & Gaps**	***Tropicoporus subindicus***	***Tropicoporus pannaensis***	***Tropicoporus xerophyticus***	***T. rudis*** **(O 915617)**	***T rudis*** **(O 915614)**	***T. stratificans*** **(VRTO884)**	***T stratificans*** **SMDB 14732**	***T. substratificans*** **(JV 1908/80)**	***T. linteus*** **(JV 0904/64)**	***T. linteus*** **(JV 0904/140)**
Tropicoporus subindicus	-	641/649(99%) 4/649(0%)	636/648(98%) 1/648(0%)	600/663(90%) 50/663(7%)	623/667(93%) 27/667(4%)	610/731(83%) 27/731(3%)	610/731(83%) 29/731(3%)	604/706(86%) 30/706(4%)	564/656(86%) 62/656(9%)	557/655(85%) 65/655(9%)
Tropicoporus pannaensis	641/649(99%) 4/649(0%)	-	632/650(97%) 5/650(0%)	596/663(90%) 50/663(7%)	617/668(92%) 27/668(4%)	604/731(83%) 32/731(4%)	604/731(83%) 34/731(4%)	599/707(85%) 56/707(7%)	559/657(85%) 62/657(9%)	552/656(84%) 65/656(9%)
Tropicoporus xerophyticus	636/648(98%) 1/648(0%)	632/650(97%) 5/650(0%)	-	589/664(89%) 51/664(7%)	612/668(92%) 28/668(4%)	600/731(82%) 26/731(3%)	600/731(82%) 28/731(3%)	594/706(84%) 29/706(4%)	557/657(85%) 63/657(9%)	550/656(84%) 66/656(10%)

Table S2. Similarities of LSU BLAST sequences of three new *Tropicoporus* species and its related species.

**Similarity % & Gaps**	***Tropicoporus subindicus***	***Tropicoporus pannaensis***	***Tropicoporus xerophyticus***	***T. rudis* (O 915617)**	***T. stratificans* (VRTO884)**	***T. substratificans* (JV 1908/80)**	***T. linteus* (JV 0904/64)**	***T. linteus* (JV 0904/140)**
Tropicoporus subindicus	-	1019/1028(99%)<br>2/1028(0%)	1020/1026(99%)<br>3/1026(0%)	830/1028(81%)<br>168/1028(16%)	797/1023(78%)<br>223/1023(21%)	1014/1349(75%)<br>5/1349(0%)	849/1037(82%)<br>179/1037(17%)	849/1030(82%)<br>169/1030(16%)
Tropicoporus pannaensis	1019/1028(99%)<br>5/1028(0%)	-	1022/1029(99%)<br>4/1029(0%)	830/1033(80%)<br>173/1033(16%)	797/1028(78%)<br>228/1028(22%)	1019/1352(75%)<br>7/1352(0%)	852/1039(82%)<br>172/1039(16%)	852/1032(83%)<br>171/1032(16%)
Tropicoporus xerophyticus	1020/1026(99%)<br>3/1026(0%)	1022/1029(99%)<br>1/1029(0%)	-	836/1067(78%)<br>171/1067(16%)	796/1026(78%)<br>226/1026(22%)	1019/1349(76%)<br>3/1349(0%)	848/1040(82%)<br>182/1040(17%)	848/1033(82%)<br>172/1033(16%)
