# Supplementary figures and images for: Characterization of New Tropicoporus Species (Basidiomycota, Hymenochaetales, Hymenochaetaceae) Discovered in Tamil Nadu, India
Source: Biology (Basel). 2024 Sep 27;13(10):770. doi: 10.3390/biology13100770 (PMC11504104; doi:10.3390/biology13100770)

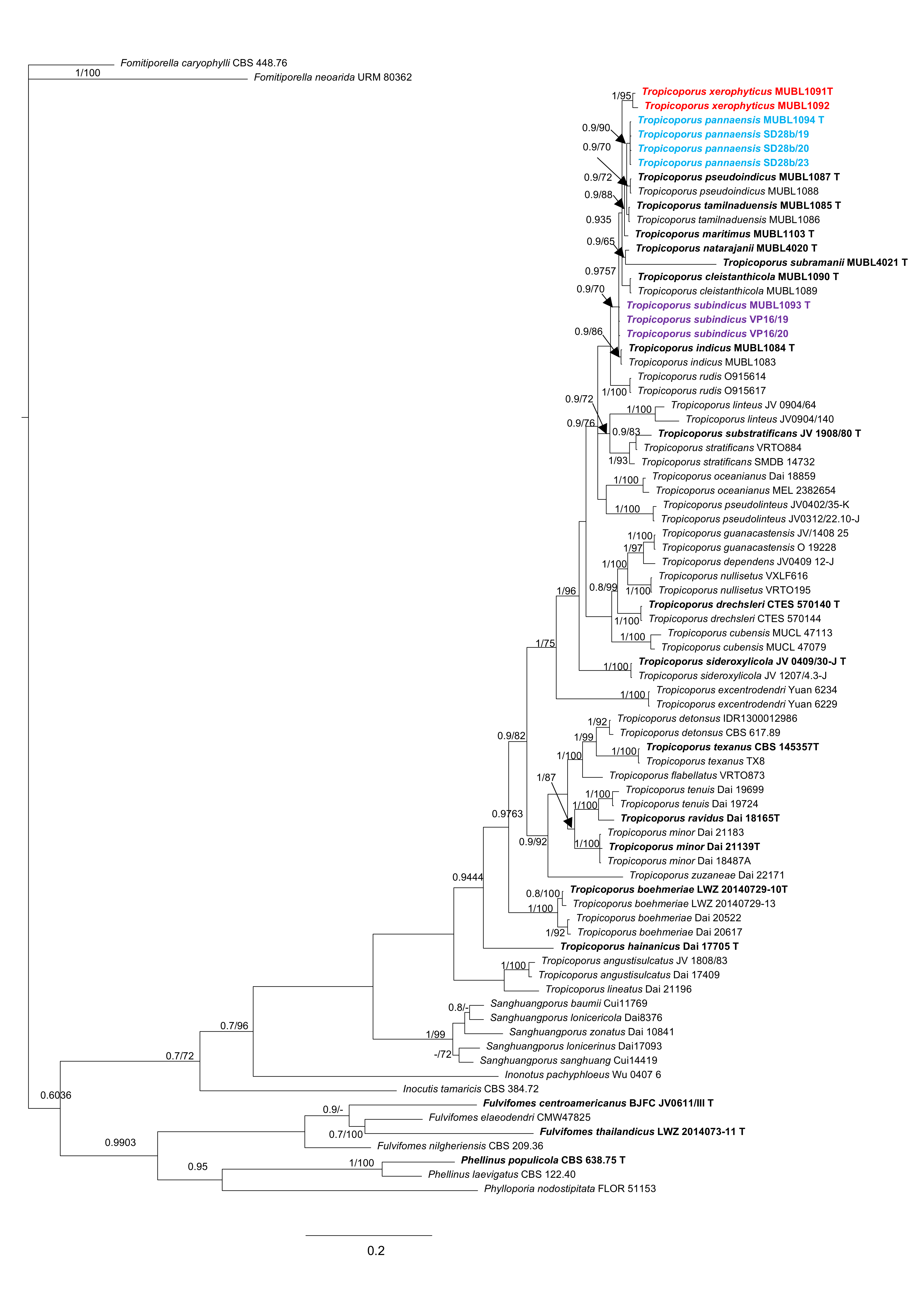

Supplement: Supplementary file 1 [file biology-13-00770-s001.zip › Supplementary Figure S1.tif]

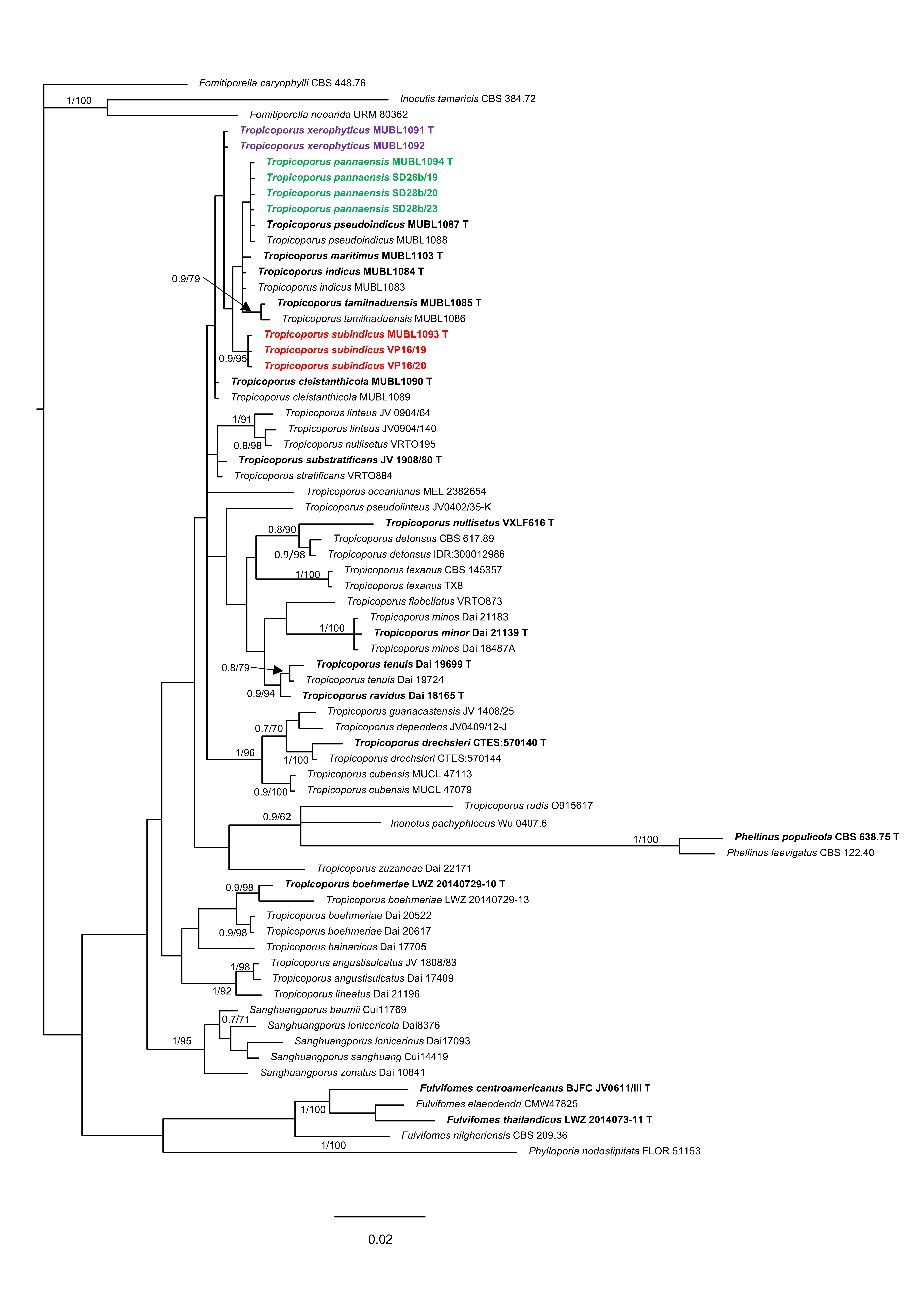

Supplement: Supplementary file 1 [file biology-13-00770-s001.zip › Supplementary Figure S2.tif]
